# Sensorimotor control of swimming *Polypterus senegalus* is preserved during sensory deprivation conditions across altered environments

**DOI:** 10.1242/jeb.245192

**Published:** 2023-05-10

**Authors:** Jeffrey Hainer, Keegan Lutek, Hailey Maki, Emily M. Standen

**Affiliations:** ^1^ Department of Biology, University of Ottawa, 30 Marie-Curie Private, Ottawa, ON, Canada, K1N 6N5; ^2^ Department of Biology, Villanova University, 800 Lancaster Avenue, Villanova, PA 19085, USA

**Keywords:** Fish, Lateral line, Vision, Viscosity, Sensorimotor, Locomotion, *Polypterus*

## Abstract

Control of locomotion involves the interplay of sensory signals and motor commands. Sensory information is essential for adjusting locomotion in response to environmental changes. A previous study using mathematical modelling of lamprey swimming has shown that, in the absence of sensory feedback, increasing fluid viscosity constrains swimming kinematics, limiting tail amplitude and body wavelength, resulting in decreased swimming speed. In contrast, previous experiments with *Polypterus senegalus* reported increased magnitude swimming kinematics (increased body curvature, body wave speed and frequency, and pectoral fin frequency) in high viscosity water suggesting that sensory information is used to adjust swimming form. It is not known what sensory systems are providing the necessary information to respond to these environmental changes. We tested the hypothesis that lateral line and visual input are responsible for the sensory-driven increase in swimming kinematics in response to experimentally increased fluid viscosity. The kinematics of five *P. senegalus* were recorded in two different viscosities of water while removing lateral line and visual sensory feedback. Unlike the mathematical model devoid of sensory feedback, *P. senegalus* with lateral line and/or visual senses removed did not reduce the magnitude of swimming kinematic variables, suggesting that additional sensory feedback mechanisms are present in these fish to help overcome increased fluid viscosity. Increases in swimming speed when both lateral line and visual sensory feedback were removed suggest that lateral line and visual information may be used to regulate swimming speed in *P. senegalus*, possibly using an internal model of predictions to adjust swimming form.

## Teaser

**Summary:** Lateral line and visual systems of *Polypterus senegalus* are not critical for responding to fluid viscosity but may be involved in a forward model of motor control to adjust locomotion.

## INTRODUCTION

Sensorimotor control is a complex process involving the use of sensory information to coordinate movement. The sensorimotor system consists of all sensory (sensory systems, afferent neurons), motor (muscles, efferent neurons), and processing (central nervous system) components involved with movement initiation and regulation. Sensory information can be sent to higher brain centres for processing, where it is used to generate signals that activate muscles to initiate or modulate movement. These brain signals can activate central pattern generators (CPGs) in the spinal cord that then produce rhythmic patterns of muscle activation (Grillner, 2006). Sensory feedback can also act reflexively on the CPGs directly without passing through higher brain centres. In both cases, sensory feedback is essential for modulation of CPGs and muscle activation patterns to control locomotion in the face of changing environmental conditions and obstacles (Grillner and El Manira, 2020). Fish are particularly interesting models to understand how multiple senses help to control locomotion because they use a variety of senses to perceive and respond to environmental stimuli (Liao, 2007; Pedraja et al., 2018; Picton et al., 2021) and they live in an aquatic environment whose physical properties can be easily manipulated. Indeed in ephemeral pools, fish may experience large changes in viscosity as water evaporates and they are forced to move through mud. Even without these natural occurrences of environmental change, viscosity is a useful tool to manipulate environmentally induced sensory feedback in swimming animals. Changing the environment a fish swims in or altering the sensory systems of a swimming fish can help give insight into how different senses contribute to the locomotor process.

Animal performance is influenced by the mechanical properties of the environment (Vogel, 1994). A single motor output will have different kinematic results in water compared with on land as a result of the constraints of the environment. Within an aquatic environment, one can change these constraints by artificially increasing the viscosity of water. By doing so, one can alter the forces and resultant sensory information experienced by a fish in a controlled way. Tytell et al. (2010) developed a computational model of a lamprey to explore how swimming performance is affected by body stiffness, muscle activation and fluid viscosity. The model consists of an actuated, elastic body (representing an elongate fish) that can simulate undulatory swimming through both internal muscular forces and external fluid forces. Notably, the pattern of muscle activation within the model is not altered in any way by the model itself. Therefore, the computational lamprey can be seen as a fish with no sensory feedback. When the viscosity of the fluid is increased in the model without altering muscle activation, a decrease in tail amplitude and wave speed resulting in a reduced swimming speed is observed (Tytell et al., 2010). The opposite kinematic response is seen in behavioural testing of living animals; swimming fish react to high-viscosity environments by increasing their lateral displacement as well as their tail and fin beat frequency to either maintain or increase swimming speed (Horner and Jayne, 2008; Lutek and Standen, 2021). This suggests that sensory feedback is used to actively adjust swimming form in viscous water, but it is as yet unclear which senses are involved in this response. Two possible sources of sensory feedback are the visual and lateral line systems.

In fish, the lateral line system uses superficial and canal neuromasts to detect flow around the body. The speed and acceleration of water flow relative to the jelly-like cupula of the neuromast cause the underlying bundles of hair cells to be displaced, resulting in action potentials being sent to the higher processing systems (McHenry and Van Netten, 2007; Windsor and McHenry, 2009). The viscosity of the external fluid medium affects lateral-line function by altering the flow around the body of a fish and therefore the deflections of the cupula (Windsor and McHenry, 2009). Thus, it is possible that feedback from the lateral line system is responsible for adjustments in swimming form in high viscosity. Fish also appear to use vision to maintain swimming speed, and to orient themselves relative to prey or obstacles (New et al., 2001; Sutterlin and Waddy, 1975). Fish maintain constant speed over the ground regardless of water flow velocity, suggesting they use visual points of reference to govern swimming speed (Standen et al., 2004). As viscosity does not remove visual cues*,* it is possible that vision may contribute to the change in swimming form in high-viscosity fluid as fish try to maintain swimming speed (or the rate of visual flow) as water viscosity increases.

In this study, we removed lateral line and visual input independently and in combination in normal and high-viscosity fluid to assess their importance in modulating locomotor control in changing environments. We used *Polypterus senegalus* as a model for these tests as they have a similar elongate body form to that of lamprey, as well as conserved components in the sensory motor system. In addition, their ability to breathe air limits the effects of viscosity on gas exchange (Couturier et al., 2007) and they display interesting changes in locomotor function across viscous environments (increased body curvature, body wave speed, body wave frequency and pectoral fin frequency; Lutek and Standen, 2021). We hypothesized that lateral line and visual input are responsible for the sensory-driven increase in swimming kinematics in response to a high-viscosity environment. We therefore expected that the loss of lateral line or vision would remove the kinematic response to viscosity, shown as a decrease in swimming speed, tail amplitude, wave frequency, pectoral fin frequency, wavelength and wave speed compared with non-sensory deprivation conditions in viscous water.

## MATERIALS AND METHODS

### Animals

*Polypterus senegalus* Cuvier 1829 were acquired from the pet trade (AQUAlity Tropical Fish Wholesale Inc., Mississauga, ON, Canada). Fish (*n*=5) (length 128.2±5.3 mm; mass 15.12±1.65 g; mean±s.e.m.) were kept in individual tanks on a 12 h:12 h light cycle at 25–26°C and fed daily. All experiments were performed according to University of Ottawa Animal Care Protocol BL-2069.

### Experimental protocol

Fish swam in a standing water tank (15 cm×83 cm) under eight different conditions and were filmed at 500 frames s^−1^ from above by two Photron Fastcam Mini UX cameras (Photron USA Inc., San Diego, CA, USA). Two cameras were used to ensure that the entire flume was in view. When necessary, custom video stitching MATLAB code (version R2018b, The MathWorks, Natick, MA, USA) was used to create a single video image between the two camera views. Videos were saved if the fish performed at least 3 steady locomotor cycles in a row, with a total of 5–10 locomotor cycles per fish in each condition. The eight conditions included all combinations of normal or blocked lateral line, 1 or 40 cP (centipoise) water, taking place in the light or dark. The order of light and viscosity treatments was randomized for each fish to minimize the effect of treatment order on kinematics. All blocked lateral line trials occurred after the normal trials to avoid possible lingering effects of the lateral line block. Methyl cellulose (400 cP; M0262, Sigma-Aldrich, St Louis, MO, USA) was added to the water to adjust viscosity to be 40 times as viscous as normal water and measured using a S2 Shell Cup^®^ (Norcross Corporation, Newton, MA, USA). At the concentration of methyl cellulose required to achieve a viscosity of 40 cP, it is expected that the solution exhibits Newtonian behaviour: viscosity remains unaffected by the deformation of the fluid (Herráez-Domínguez et al., 2005). The flume was lit from below, using three LED lights for light trials and three infrared lights for dark trials. Infrared light was assumed to be invisible to *P. senegalus*, as with other nocturnal fishes (Fitzpatrick et al., 2013). Light was directed at a mirror and diffused through white acrylic into the flume from below. All other sources of light besides experimental sources were eliminated. Fish remained in these viscosity conditions for no more than 10 min at a time to minimize stress and prevent injury to fins.

Lateral line block was achieved using a 0.15 mmol l^−1^ cobalt (II) chloride (Sigma-Aldrich) solution in calcium-free fresh water. Calcium-free fresh water was prepared according to the procedure outlined by Karlsen and Sand (1987). The *P. senegalus* test subject was submerged in solution for 3 h. After 3 h, the fish was placed in aquarium water for 5 min prior to trials to rinse off excess cobalt (II) chloride solution. Because even with an intact lateral line *P. senegalus* do not reliably respond to an escape stimulus, the success of the lateral line block was confirmed by staining the lateral line neuromasts with the fluorescent dye 4-(4-diethylaminostyryl)-1-methylpyridinium iodide 4-Di-2-ASP (4-Di-2-ASP). The fish was then anaesthetized in a solution of MS-222 (200 mg l^−1^) and visualized using a ZEISS Axio Zoom V16 microscope. Darkened lateral line neuromasts compared with a control fish with an intact lateral line system was indicative of block success (Fig. S1).

### Kinematic processing and variables

A neural network created using the markerless pose estimation program DeepLabCut (Nath et al., 2019) was used to digitize the nose, tail and fins of the fish in each video (Movie 1). DeepLabCut data were converted to be compatible with DLTData Viewer 6 (Hedrick, 2008). Data files were fixed manually using DLTData Viewer 6 in cases where neural network digitized points were placed incorrectly. Then, to remove jitter from the neural network digitized body and fin points, these points were filtered with a low-pass filter of 5 times body and pectoral fin frequency, respectively. Videos were binarized in FIJI (Schindelin et al., 2009) and custom MATLAB code was used to calculate midlines and magnitude variables for each trial, based on binarized images and head and tail points.

The following magnitude variables were extracted: swimming speed (body lengths per second, BL s^−1^), pectoral fin frequency (Hz), pectoral fin state, tail amplitude (BL), body wave frequency (Hz), body wave speed (%BL s^−1^), and body wavelength (BL). Pectoral fin frequency was defined as the number of fin beat cycles per second, with a fin beat cycle being characterized by pectoral fin movement between two consecutive instances of fin adduction of the right pectoral fin. If no pectoral fin beats occurred during a trial, pectoral fin frequency was not recorded. Pectoral fin state was used to denote whether a fish did or did not use their pectoral fins during a trial. Fin use was designated ‘On’, and lack of fin use was designated ‘Off’ for each trial. Tail amplitude was defined as the perpendicular distance between an intervening maximum tail amplitude on the right side of the fish and a line drawn between two consecutive maximum tail amplitudes on left side of the fish, divided by two. Body wave frequency was defined as the number of locomotor cycles per second, with a locomotor cycle being characterized by the motion between two consecutive maximum tail amplitudes on the same side of the fish. Body wave speed was calculated as the speed of a wave of curvature as it travelled from 75% to 95% BL along the fish. Body wavelength was calculated as the linear distance between consecutive instances of minimum or maximum curvature.

### Statistical analyses

Statistics and graphing were carried out in R 3.6.1 (http://www.R-project.org/). Linear models were used to infer which independent variables influence the kinematic variables. Each variable was fitted to a suitable linear model with mixed effects, created using the R package nlme (https://CRAN.R-project.org/package=nlme). Swimming speed, lateral line condition, light condition and viscosity were used as fixed effects while individual was treated as a random effect. Unequal variances were corrected using a constant variance function (varIdent) where applicable as in Lutek and Standen (2021). Multiple comparisons were performed across treatments using the estimated marginal means of each model (https://CRAN.R-project.org/package=emmeans). To control for the false discovery rate, the Bonferroni correction was applied based on the number of comparisons performed on a variable-to-variable basis. Significance of Bonferroni-corrected *P*-values was defined as those cases where *P* was less than 0.05. Graphs were created using ggplot2 (Wickham, 2016). A two-sided Fisher's exact test was performed on pectoral fin state count data to assess differences across conditions (Table S1). In one trial that occurred in normal water with no sensory deprivation, swimming speed was 23.5% faster than in the next fastest trial. Removing this trial did not change any of the results for the multiple comparisons but did result in normality of residuals for the model. Even though linear mixed models are robust to violations of residual normality (Knief and Forstmeier, 2021), the trial was removed as an outlier.

The dataset and R code used for statistical analysis are included as supplementary files (Table S2, Supplementary Materials and Methods). Additional data and code are available upon request.

## RESULTS

**Fig. 1.**
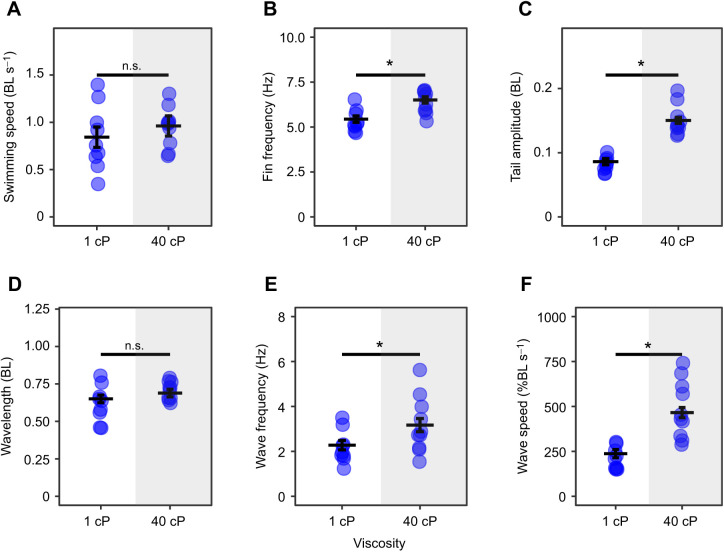
**The effect of increased viscosity on swimming kinematics in *Polypterus senegalus*.** Swimming speed (A), pectoral fin beat frequency (B), tail amplitude (C), body wavelength (D), body wave frequency (E) and body wave speed (F) at normal (1 cP, white shading) and high (40 cP, grey shading) viscosity are shown (BL, body lengths). Estimated marginal means based on a linear model with mixed effects are represented by horizontal lines, including error bars (±s.e.m.). *n*=5 for each treatment, 2 trials per fish within a treatment, each trial represented by a circle. Black bars represent comparisons between conditions. *Significant difference (n.s., non-significant difference).

**Fig. 2.**
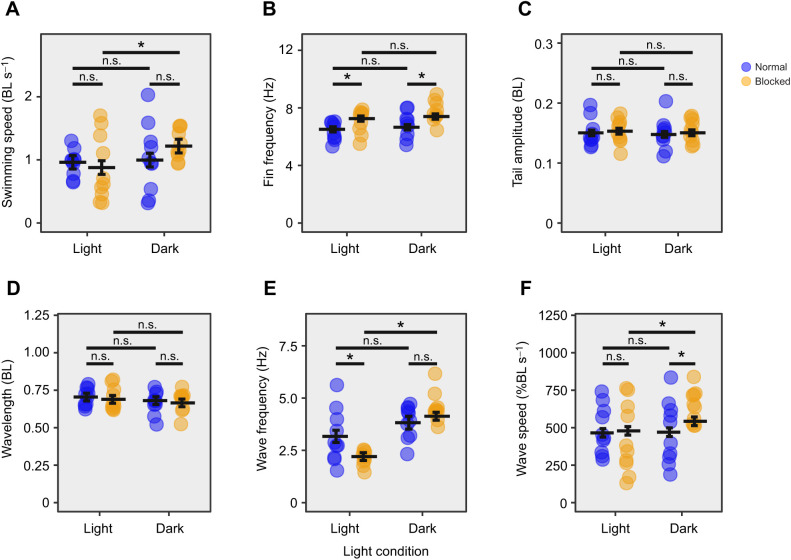
**The effect of sensory deprivation conditions in high-viscosity water on swimming kinematics.** Swimming speed (A), pectoral fin beat frequency (B), tail amplitude (C), body wavelength (D), body wave frequency (E) and body wave speed (F) at high viscosity (40 cP) in the light and dark, with and without lateral line block are shown. Estimated marginal means based on a linear model with mixed effects are represented by horizontal lines, including error bars (±s.e.m.). *n*=5 for each treatment, 2 trials per fish within a treatment, each trial represented by a circle. Black bars represent comparisons between conditions. *Significant difference (n.s., non-significant difference).

**Fig. 3.**
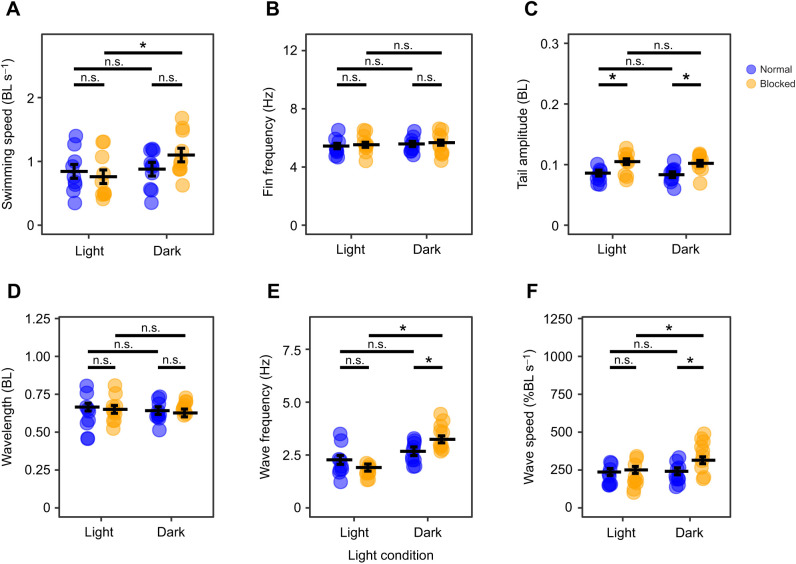
**The effect of sensory deprivation conditions in normal water on swimming kinematics.** Swimming speed (A), pectoral fin beat frequency (B), tail amplitude (C), body wavelength (D), body wave frequency (E) and body wave speed (F) in normal water (1cP) in the light and dark, with and without lateral line block are shown. Estimated marginal means based on a linear model with mixed effects are represented by horizontal lines, including error bars (±s.e.m.). *n*=5 for each treatment, 2 trials per fish within a treatment, each trial represented by a circle. Black bars represent comparisons between conditions. *Significant difference (n.s., non-significant difference).

Fish swam with a similar range of speeds in both normal and high-viscosity water (Fig. 1A). Swimming speed was a significant predictor of all kinematic variables (Table 1). An increase in viscosity elicited an increase in pectoral fin frequency, tail amplitude, wave frequency and wave speed compared with swimming in regular water (Fig. 1B,C,E,F). Apart from a slight decrease of wave frequency when the lateral line was blocked, sensory deprivation did not illicit the expected decrease of swimming kinematics in viscous water (Fig. 2); however, lateral line condition, light condition and the interaction between lateral line and light conditions were significant predictors of swimming speed (Table 1). No matter the water condition, when lateral line and visual sensory input were removed together, there was an increase in swimming speed (Figs 2A, 3A). Lateral line condition and the interaction between lateral line condition and viscosity were significant predictors of pectoral fin frequency (Table 1), indicating that removal of the lateral line increases pectoral fin frequency in high-viscosity environments but not normal water (Figs 2B, 3B). Lateral line condition, and the interaction between viscosity and lateral line condition were significant predictors of tail amplitude (Table 1), indicating that removal of the lateral line increases tail amplitude in water but not in high-viscosity environments (Figs 2C, 3C). Light condition did not affect pectoral fin frequency or tail amplitude (Table 1). There was no association between trial condition and the pectoral fin state (Table S1). A full list of pairwise comparisons can be found in Tables 2–4.

**Table 1.**
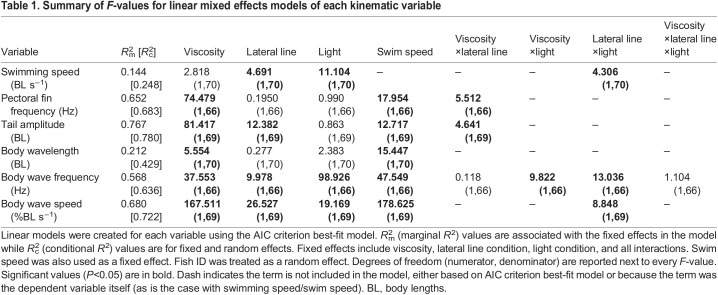
Summary of *F*-values for linear mixed effects models of each kinematic variable

**Table 2.**
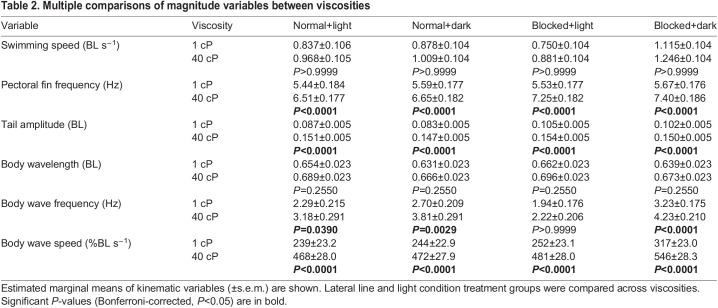
Multiple comparisons of magnitude variables between viscosities

## DISCUSSION

### Kinematic response to viscosity

In accordance with previous studies (Horner and Jayne, 2008; Lutek and Standen, 2021), fish with intact sensory systems altered their swimming kinematics when swimming in viscous water (Fig. 1B,C,E,F). Based on the importance of the lateral line and vision in the sensory control of fish swimming (Liao, 2006; Standen et al., 2004; Sutterlin and Waddy, 1975), and on mathematical modelling that predicts a lower magnitude of swimming kinematics in the absence of sensory input (Tytell et al., 2010), we predicted that removing the lateral line and vision would elicit a reduced magnitude of swimming kinematics in viscous water. In fact, our results suggest that the removal of lateral line and visual sensory information independently and in combination did not decrease the magnitude of swimming kinematic variables in viscous water (Fig. 2; note body wave frequency exception discussed below). Conservation of kinematics when these systems are compromised suggests that, in opposition to our hypothesis, the lateral line and visual systems are not solely responsible for sensory feedback control of high-viscosity locomotion in *P. senegalus*. In this case, additional sensory inputs or feed-forward control mechanisms must be essential in these fish.

A potential source of sensory feedback that could be used in steady swimming and may respond to altered environmental conditions are stretch-receptive cells. While lateral line and visual systems project to supraspinal centres that modulate spinal circuits via high-level commands (Bollmann, 2019; Thandiackal et al., 2021), intraspinal stretch-receptor neurons can directly modulate spinal reflexes essential for locomotion without input from the brain centres (Hsu et al., 2017). Stretch-receptive cells are present in the muscle of rays (Fessard and Sand, 1937) as well as along the spinal cord of lampreys and zebrafish (Grillner et al., 1991; Picton et al., 2021). Such stretch-receptive cells can detect lateral body movements during swimming, and modulate locomotion by affecting rhythm-generating interneurons in the spinal cord (Picton et al., 2021). Although the existence of stretch receptors has yet to be confirmed in *P. senegalus*, the ability to encode body curvature from the degree of stretch could facilitate the muscle activation required to maintain swimming speed in different environments. In viscous water, similar intensities of muscle activity that would be utilized in normal water would result in less local bending (Tytell et al., 2010). Reduced local bending could be encoded by stretch receptors and could be used to signal an increase in motor effort to achieve the larger amplitude kinematic outputs we see when fish compensate for high viscosity. Of course, other yet to be discovered modalities may also be present acting reflexively or through integration in higher brain centres to fine-tune sensory feedback.

### Higher order integrative process

Multiple sensory modalities can provide information used in a single behaviour, forming a complex network of interacting sensory systems. In this study, loss of the lateral line at high viscosity was correlated with an increase in pectoral fin beat frequency (Fig. 2B). The lateral line is thought to map the pattern of flow conditions surrounding the fish (Sutterlin and Waddy, 1975; Windsor and McHenry, 2009). Removing lateral line information reduces information about stability requirements in the environment. The increase in pectoral fin beat frequency may be a mechanism that allows the use of mechanosensory capacities in the fin to identify swimming stability needs. In this way, the increased fin beat frequency may be compensating for the lack of information coming from body lateral line organs that have been blocked. The lateral line system appears to be integrated with information from other modalities in other regions of the fish to control locomotion. Although multiple modalities may provide similar information about a single stimulus, the neural circuitry and resultant integration process of each sense can differ (Stein et al., 2014). Cross-modal integration of different sensory signals allows for the modulation of movement depending on the information available from each sense, as well as the relationship between the separate sensory tracts of similar information.

When both lateral line and visual sensory input were removed, there was an increase in swimming speed (in both normal and high viscosity; Figs 2A, 3A). Similar increases in swimming speed were seen when lateral line input was removed in blind cave fish and when both lateral line and vision were removed in closely related river fish (Hassan et al., 1992). These changes were attributed to attempts at increasing lateral line sensory feedback by increasing flow across the body. Interestingly, this conclusion assumes that muscle effort is being altered to obtain an expected level of sensory feedback. The modulation of muscle effort to alter sensory feedback in this goal-oriented manner suggests the existence of a theoretical forward model of motor control: an integrative model that uses the comparison between the internally predicted sensory results of muscle activation and the actual sensory outcomes to modulate movement (Welniarz et al., 2021). This forward model relies on the discrepancies between predicted sensory consequences of an action provided by feed-forward loops (i.e. higher order signals) and the actual information received during an action provided by feedback loops (i.e. signals from the environment) (Popa and Ebner, 2019; Welniarz et al., 2021). These discrepancies, called prediction errors, are then used to adjust muscle activity to accomplish the desired behavioural goal. When lateral line and visual input are missing, the disparity between this missing input and the expected sensory input could result in prediction errors, falsely signalling to the fish that it has not yet reached a target speed. The motor command may then be altered to increase muscle activation, resulting in an increase in swimming speed that exceeds the desired goal. While it is unclear whether *P. senegalus* has this forward model of motor control, both lateral line and visual systems appear to be involved with the generation of prediction errors in other species of fish (Ryu and Kuo, 2021; Straka et al., 2018; Torigoe et al., 2021). Evidently, locomotor control is dependent on the information available to an organism as well as the integration systems at hand.

### Body wave parameters

While sensory deprivation did not decrease most kinematic variables in viscous water, removing only lateral line sensory input in high viscosity led to a decrease in body wave frequency (Fig. 2), suggesting that the lateral line sensory feedback regulates body wave frequency. The lateral line system appears to provide feedback about the fish's own movement (Ayali et al., 2009). Furthermore, feedback between sequential lateral line neuromasts has been theorized to allow the calculation of body wave parameters such as wave frequency (Skandalis et al., 2021). The body wave parameters encoded by sequential neuromasts could be altered or missing when the lateral line is blocked, resulting in changes in wave frequency as the fish is unable to determine its own movement.

This reduction in wave frequency could also be a result of interactions between multiple sensory systems and the previously discussed theoretical forward model of motor control. Previous experiments demonstrated that when the lateral line is blocked but vision remains intact, some kinematics of *P. senegalus* increase in variability, such as body wavelength (Znotinas, 2018). It was proposed that a sensory mismatch occurred, where the lateral line and sight were reporting conflicting information, resulting in altered kinematics. A mismatch between different sensory modalities could be considered another broader version of prediction error where the difference between sensory inputs is so great that the animal cannot determine a specific ‘goal’ but modifies its behaviour erratically, resulting in high kinematic variation. As the reduction of wave frequency seen in our work occurred during a possible conflict of lateral line and visual information, the mismatch between the sensory systems could be driving the changed kinematics.

### Mechanical constraint

Mechanical constraint exists in all biological systems. It is important to understand how these constraints affect movement outside of any changes in muscle effort. If significant enough, mechanical constraint can limit the kinematic output of a given motor signal. For example, increasing the force with which to push against a solid wall would increase motor effort without changing kinematic output. We propose that the increased mechanical constraint in viscous environments can explain the differences in kinematic response between water and high-viscosity environments we see in our data. When the lateral line is blocked in normal water, an increase in tail amplitude is observed (Table 3, Fig. 3C). One would expect a similar response by the fish regardless of water viscosity; however, blocking the lateral line in high viscosity yielded no increase in tail amplitude (Table 3, Fig. 2C). The lack of tail amplitude increase may have been due to the increase in mechanical constraint of high-viscosity fluid overpowering the effects of muscle activation levels. In other words, the increase in viscosity may limit the kinematic output of the muscles independent of muscle activation signal. To investigate this further, muscle activity could be monitored between blocked and intact lateral line treatments to see whether there is any difference in muscle activation. Greater muscle activation with a constant tail amplitude magnitude would suggest that mechanical constraint is limiting the system.

**Table 3.**
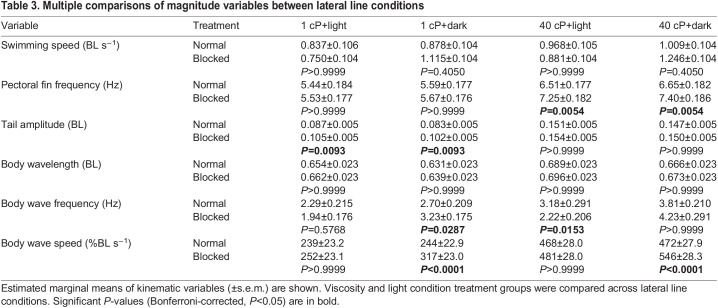
Multiple comparisons of magnitude variables between lateral line conditions

**Table 4.**
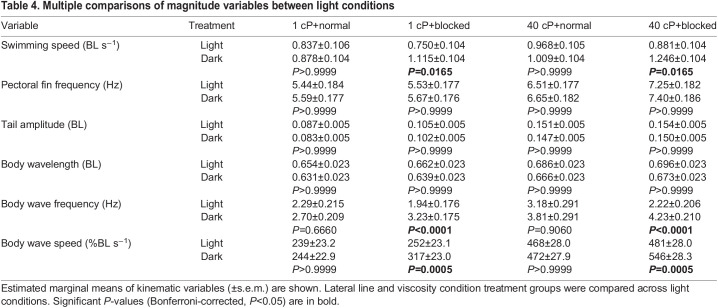
Multiple comparisons of magnitude variables between light conditions

### Conclusion

We propose that *P. senegalus* use multiple sensory feedback modalities and control strategies to navigate their environments. Preservation of neural control in the absence of lateral line and/or visual sensory feedback suggests that other sensory systems, potentially stretch-receptive cells as found in lamprey and zebrafish, help *P. senegalus* respond to novel environmental mechanical constraints. We also propose that regardless of viscosity, lateral line and visual sensory feedback modulate aspects of undulatory locomotion such as swimming speed, possibly using prediction errors and a feed-forward model of motor control. Finally, mechanical constraint limits changes in kinematics, regardless of sensory feedback. These results show that changing environmental viscosity as well as the sensory systems available to a fish can be used to gain insight into the integration process, and how information from multiple sensory modalities is used to modulate locomotion.

## Supplementary Material

10.1242/jexbio.245192_sup1 Supplementary information
