# Supplementary material for: Sensorimotor control of swimming Polypterus senegalus is preserved during sensory deprivation conditions across altered environments
Source: J Exp Biol. 2023 May 10;226(9):jeb245192. doi: 10.1242/jeb.245192 (PMC10184767; doi:10.1242/jeb.245192)
Supplement: Supplementary information [file jexbio-226-245192-s1.pdf]

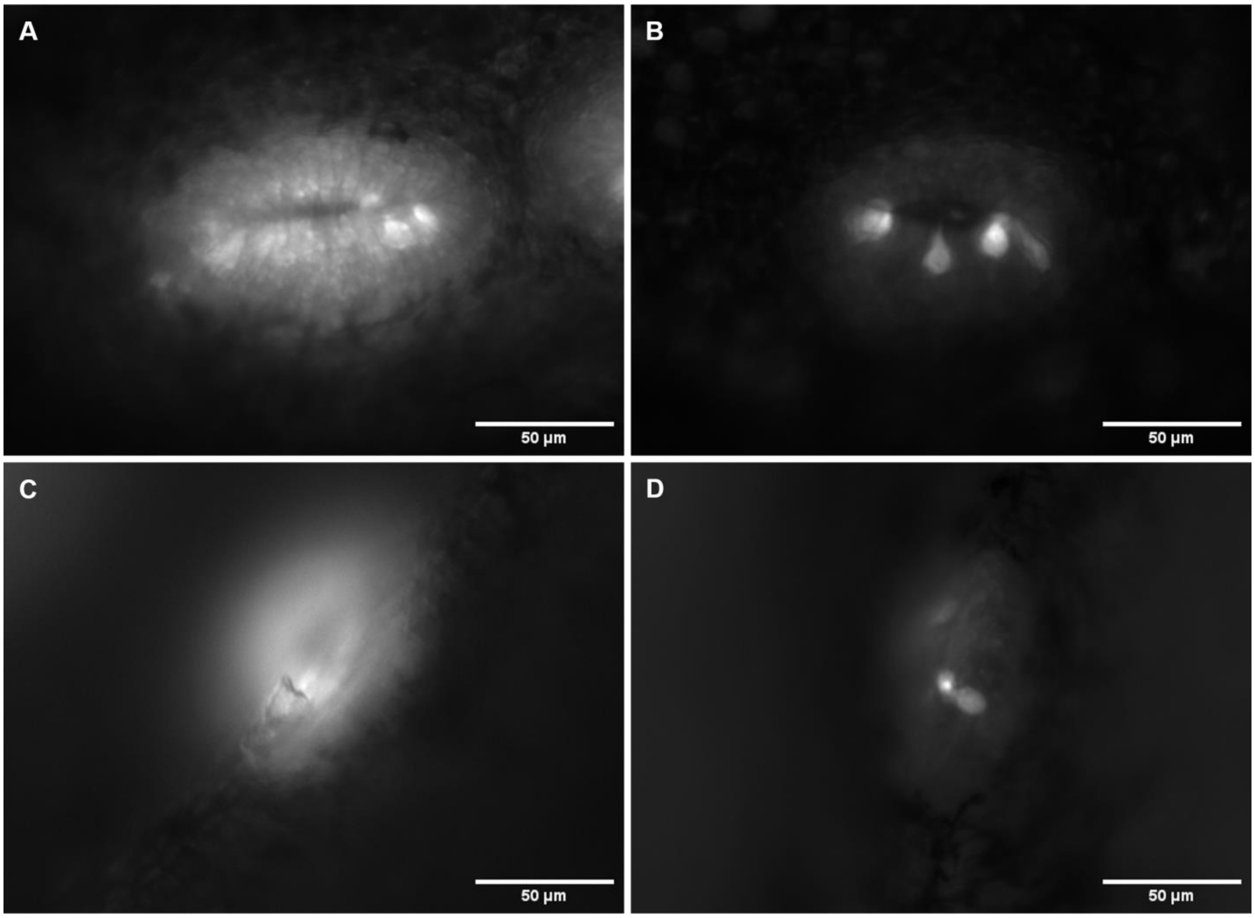

Fig. S1. Comparison of lateral line neuromast activity, visualized under a ZEISS Axio Zoom.V16 fluorescence microscope. Lateral line neuromasts were stained with the fluorescent dye, 4-(4-diethylaminostyryl)-1methylpyridinium iodide 4-Di-2-ASP (4-Di-2-ASP). Normal canal neuromasts (A), cobalt-treated canal neuromasts (B), normal pit neuromasts (C), and cobalt-treated pit neuromasts (D) are shown. 4-Di-2-ASP is taken up by the ionic transduction channels of functioning lateral line neuromasts, resulting in greater fluorescence in normal neuromasts (A,C) compared to cobalt-treated neuromasts (B,C). Micrographs of normal canal neuromasts (A,C) and cobalt-treated neuromasts (B,D) are from two separate fish at identical light intensities (7 %) and exposures (70 ms).

Journal of Experimental Biology • Supplementary information

Table S1. Fin state count data presented in contingency table with results for Fisher's Exact Test. Trials designated as "On" or "Off" for each condition were counted, with total counts being entered into the appropriate cell of the contingency table. A two-sided fisher's exact test was performed to determine if counts were dependent on trial condition. As the p-value from Fisher's Exact Test is greater than 0.05, we cannot reject the null hypothesis that there is no association between condition during the trial and the fin state.

**Condition**			**Fin State Counts**			**Fisher's Exact Test p-value**
Viscosity	Lateral Line	Light	Fins On	Fins Off	**Totals**	
1 cP	Normal	Light	9	0	9	p > 0.9999
1 cP	Normal	Dark	10	0	10	
1 cP	Blocked	Light	10	0	10	
1 cP	Blocked	Dark	10	0	10	
40 cP	Normal	Light	10	0	10	
40 cP	Normal	Dark	9	1	10	
40 cP	Blocked	Light	9	1	10	
40 cP	Blocked	Dark	9	1	10	
	**Totals**		76	3	79	

Table S2. Trial means for each kinematic variable. Full set of trial means used in statistical analysis, with each row containing the conditions and means for a different trial. Columns include fish name (fish), trial number (trial), lateral line condition (LL), light condition (light), swimming speed (ss), pectoral fin state (FinState), pectoral fin frequency (FinFreq), tail amplitude (TailAmp), body wavelength (WaveLength), body wave frequency (WaveFreq) and body wave speed (WaveSpeed).

Click here to download Table S2

Supplementary Materials and Methods - R Markdown code for statistical analysis

R-script formatted using the R package rmarkdown (https://CRAN.R-project.org/package=rmarkdown). Includes code for Fisher's exact test for pectoral fin state, summaries of linear mixed models, parametric tests of models (normality of residuals and equality of residual variance across treatments), unequal variance corrections where needed, estimated marginal means, and Bonferroni corrected multiple comparisons.

Click here to download Rcode

Journal of Experimental Biology • Supplementary information

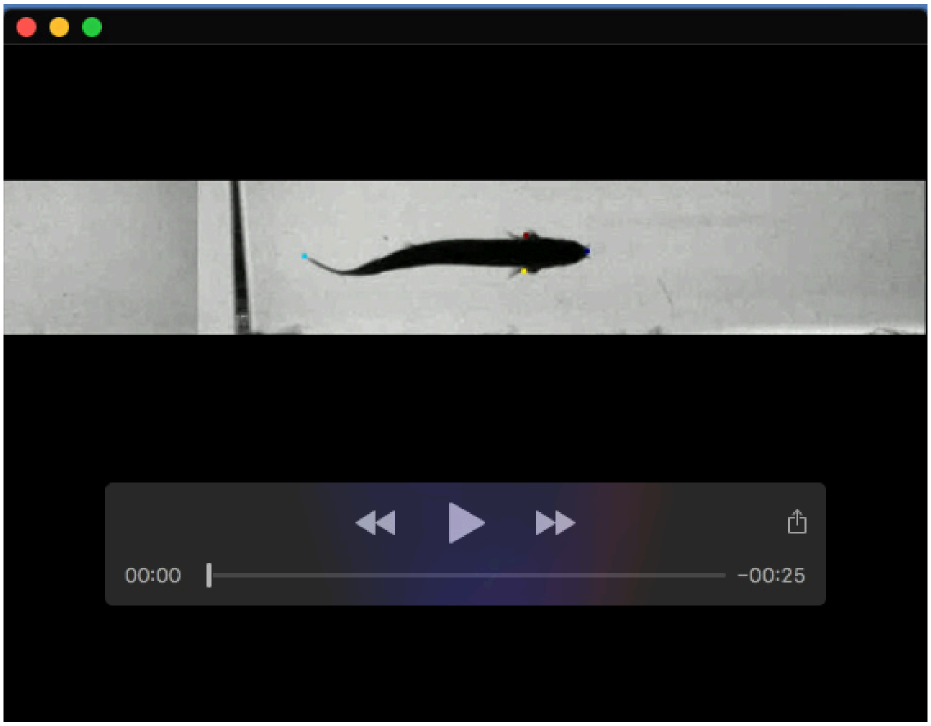

Movie 1. Video example of DeepLabCut point tracking on swimming *P. senegalus*. Fish was filmed swimming in normal water in the dark at 500 frames per second. Nose (purple), tail (blue), right pectoral fin (yellow) and left pectoral fin (red) were tracked in each frame using the markerless pose estimation program, DeepLabCut.

Journal of Experimental Biology • Supplementary information
